# Patients' perceptions of safety and quality of maternity clinical handover

**DOI:** 10.1186/1471-2393-11-58

**Published:** 2011-08-10

**Authors:** Georgiana SM Chin, Narelle Warren, Louise Kornman, Peter Cameron

**Affiliations:** 1 Centre for Research Excellence in Patient Safety, Department of Epidemiology and Preventive Medicine, Monash University, Australia; 2 The Royal Women's Hospital, Parkville, Victoria, Australia; 3 School of Psychology and Psychiatry, Monash University, Australia; 4 Department of Obstetrics and Gynaecology, University of Melbourne, Australia

## Abstract

**Background:**

Maternity clinical handover serves to address the gaps in knowledge existing when transitions between individuals or groups of clinicians occur throughout the antenatal, intra-partum and postnatal period. There are limited published studies on maternity handover and a paucity of information about patients' perceptions of the same. This paper reports postnatal patients' perceptions of how maternity handover contributes to the quality and safety of maternity care.

**Methods:**

This paper reports on a mixed-methods study consisting of qualitative interviews and quantitative medical record analysis. Thirty English-speaking postnatal patients who gave birth at an Australian tertiary maternity hospital participated in a semi-structured interview prior to discharge from hospital. Interview data were coded thematically using the constant comparative method and managed via NVivo software; this data set was supplemented by medical record data analysed using STATA.

**Results:**

Almost half of the women were aware of a handover process. Clinician awareness of patient information was seen as evidence that handover had taken place and was seen as representing positive aspects of teamwork, care and communication by participants, all important factors in the perception of quality health care. Collaborative cross-checking, including the use of cognitive artefacts such as hand held antenatal records and patient-authored birth plans, and the involvement of patients and their support people in handover were behaviours described by participants to be protective mechanisms that enhanced quality and safety of care. These human factors also facilitated team situational awareness (TSA), shared decision making and patient motivation in labour.

**Conclusions:**

This study illustrates that many patients are aware of handover processes. For some patients, evidence of handover, through clinician awareness of information, represented positive aspects of teamwork, care and communication.

Cross-checking and cognitive artefacts were observed to support handover. Patient-authored birth plans were described by some to enhance the quality and safety of the handover by providing a 'voice' to the patient in this process. This was a novel and potentially important perspective.

Future research involving patients and their support people in supporting and evaluating handover should be considered.

## Background

Maternity care predominantly involves the care of healthy, low-risk women and their babies for a defined period of normal physiologic change (pregnancy, birth and transition to the non-pregnant state). However, this is not always the case, and levels of risk are influenced by medical conditions that pre-exist or develop during the pregnancy (which may or may not be pregnancy-related); psychosocial factors may also be important contributors to the level of risk [1-3]. Care is focused on the woman and her baby but may also extend to involve the wider family and support people, particularly during labour.

Transitions of care occur throughout the whole continuum of pregnancy. They occur between multiple health professionals antenatally (such as occurs in shared antenatal care, midwifery-led models of care and between outpatient departments), intra-partum (within labour) and in transitions of care between wards, theatre and the community on post-natal discharge from hospital. Transitions in care may also occur during inter-hospital transfers when additional external specialist care is required [1,2,4,5].

Within and between shifts, particularly in the acute setting of birth suite, the team of clinicians required to care for each patient may constantly change in terms of number, speciality and seniority depending on the needs of the woman and her newborn. Communication is paramount to enhance the safety and quality of these transitions [6].

By design, the clinical handover is a process that exists to do this job. It is the formalised transfer of responsibility and accountability between individual and teams of clinicians for all or some aspects of care for one or more patients on a permanent or temporary basis [7]. Information sharing and ensuring that this information is understood by oncoming staff is an essential part of this process [8]. Issues with this process may result in adverse outcomes [9,10].

Some studies have been conducted specifically on maternity handover and within the wider context of professional communication in maternity care [5,6,11]; several have also embedded their discussion of maternity handover within an analysis of handover in other healthcare contexts [12-14]. Observed strategies to enhance quality and safety of handovers have their roots within human factors, a term which originates from human engineering and refers to a property specific to humans which may impact upon the interpersonal and physical environment [15]. The use of standardisation, cognitive artefacts (articles to enhance cognition and memory) such as whiteboards and other written documents, team situational awareness (TSA, whereby each team member has a shared perception, comprehension and projection of the current situation affecting themselves, environment and task/s) and the presence of a team co-ordinator have all been cited as interlinked elements that enhance patient safety [5,6,10,11,13,14]. All these elements are part of distributed cognition where overlapping and shared knowledge between individuals and teams through physical and non-physical routes facilitates co-ordination and teamwork [16,17]. Communication within the Birth Suite is the key area which has been studied in maternity care handover, with minimal focus on the ward or antenatal setting. There is a paucity of information of the patient perception of maternity clinical handover. In particular, the quality and safety, and patient involvement in handover have not been investigated. This exploratory study investigates postnatal patients' perceptions of maternity handover and factors that affect the quality and safety of this process. It is hoped that such information will be able to inform future handover improvements from a patient's perspective.

## Methods

This paper reports on a mixed methods study consisting of qualitative interviews and quantitative medical record analysis. Thirty English speaking women aged 18 and over who gave birth at an Australian tertiary maternity hospital in 2007 were invited and participated in a single semi-structured interview. They all provided signed informed consent to be in the study prior to interview which also included consent to access their medical record data. One additional woman initially agreed to participate but later withdrew consent prior to interview.

Another 35 women were invited to participate but declined consent, six others were discharged from hospital before consent could be obtained, and four were ineligible to be recruited for this study.

At the time of data collection, in-patient maternity care within this hospital comprised three Birth Suites (including one midwifery-led Birthing Unit), two postnatal wards and one antenatal ward. The model of maternity care was divided into low and high maternity risk units, and also included the provision of shared antenatal care with registered community general practitioners [18,19].

A list of all births in ascending patient unit record number over the 24 hour period (0900 to 0859) was obtained each study day and every third patient was identified to be approached for recruitment. Women aged less than 18 years, were non-English-speaking or who had been treated by the researchers were excluded from study. Potential participants were approached by the first author within 24 hours following birth of their babies and were interviewed prior to discharge two to five days post-birth. Printed information was given; participants were given time to read this and provided an opportunity to ask questions before signing their consent form. Recognising the relative vulnerability of participants given their in-patient status and recent childbirth, and to enhance engagement with the researcher, interviews were not audio-recorded; instead, notes were taken during each interview and expanded in detail immediately after the interview concluded [20,21]. Interview data was supplemented by demographic and obstetric data obtained, with consent, from patient medical records. All data was de-identified to maintain participant confidentiality.

During interviews, participants were asked questions to collect demographic data before specific study questions were explored. Topics covered included: childbirth education, patients' understandings of perinatal events and care, their awareness of handover, perceptions of content and setting of handover, and suggestions for improvement of maternity handover. The questions were designed to firstly ascertain whether patients were aware of handover and explaining how they knew handover took place. Further questions were then asked to explore their views on what was acceptable and safe practice. The inclusion of demographic, childbirth education and clinical history questions were chosen to illustrate the background of the patients we were studying.

Interviews were scheduled at a time during the participant's hospital stay so that she could reflect upon her whole period of hospital-associated care, from the booking visit to the immediate postnatal admission. This was both a reasoned and pragmatic decision. A single interview enabled the participant to give their fresh ideas about this process when first introducing to them the concept of 'handover'. This also eliminated the effect multiple interviews may have in influencing change in the patient's behaviour in interactions with clinical staff and the flow on effect on their perceptions of care.

Interview notes were coded thematically by the first author using the constant comparative method [22], whereby themes that were identified in one interview were searched for in subsequent interviews; when an emergent theme was identified, the researcher revisited the previously analysed notes to check for its presence. This approach, derived from grounded theory [23], enabled an additional level of rigor within the data analysis. In this paper we also report on themes that were mentioned by only a few participants but which have clinical relevance. All themes were discussed with the second author. Consensus was reached following further discussion and secondary data review with the second author when any ambiguity or disagreement occurred. Qualitative data was analysed using NVivo software. Medical records data were analysed using STATA.

Institutional ethics approval for this project was obtained from The Royal Women's Hospital Human Research Ethics Committee (Melbourne, Australia). This study also complies with the Helsinki Declaration [24].

## Results

### Demographics and clinical information

The majority of the participants in this study were in-patients following the birth of their first child (Table 1) and this was first personal experience of maternity handover. All gave birth at 34 or more weeks' gestation, with the majority giving birth around term. The majority received their care in either a low risk obstetric unit or through a low risk midwifery model of pregnancy care in the Family Birth Centre. Almost a third of participants were involved in a shared model of antenatal care [25], where they received their antenatal care from a general practitioner (family physician) in collaboration with hospital obstetric staff. Most had a vaginal birth and all had attended some formal childbirth education during their pregnancy, as well as using other, self-obtained pregnancy and/or parenting information.

**Table 1:** Demographics and obstetric information (N = 30)

Characteristic	Number	Percent
**Age (range 20-42 years)**		
> 40 years	1	3.3%
31-40 years	17	56.7%
20-30 years	12	40%
**Parity**		
Primiparous	21	70%
Multiparous	9	30%
**Gestation at birth (range 34-42 weeks)**		
29-30 weeks (intermediate risk pre-term)	2	6.6%
37-41 weeks (full-term)	26	86.7%
42+ weeks (post-dates)	2	6.6%
**Relationship status**		
Married/De-facto	28	93.3%
Separated	1	3.3%
Single	1	3.3%
**Patient's country of birth**		
Australia	21	70%
Outside Australia	9	30%
**Highest level of education attained**		
Post-graduate education	5	16.6%
Tertiary (undergraduate education)	19	63.3%
Completed secondary education, or equivalent	5	16.6%
Some secondary education	1	3.3%
**Clinical Unit**		
'High Risk' Obstetric Unit	7	23.3%
'Low Risk' Obstetric Unit	18	60%
'Low Risk Obstetric', midwifery-led Birth Centre	5	16.7%
**Shared Care with General Practitioner**	8	26.7%

### Handover awareness and quality in maternity handover

Almost half (47%; n = 14) of the participants described having some awareness of a handover process prior to being invited to take part in the study, with 8 (27%) having experienced this through the shared care model of antenatal visits. Once the concept of handover was introduced to those who were previously unaware of handover, all reported instances of handover. The most frequently reported evidence that handover had occurred were: clinician's awareness of information about a patient which they had not previously discussed with that patient (57%; n = 17), patient being present during a verbal handover (50%; n = 15), the existence of documentation (e.g. a hand-held patient record) as an example of written handover (36%; n = 11), and patients' awareness of shift changes (23%; n = 7).

Clinicians' awareness of patient information was interpreted by some women as representing positive aspects of teamwork, care and communication (i.e. representing consistency of team care and communication, efficient flow of work and professional conduct), and was reported in this way by five (17%) women. One woman also reflected that the way a lactation consultant spoke to her represented an awareness of her case pre-dating their discussion review. She reported that this gave her the feeling that this clinician understood her current problems.

One-third of participants specially mentioned that handovers were done to their satisfaction or better. These women referred to the idea of a "good" handover as those that represented that clinicians were doing a good job (13%; n = 4), resulted in patients having a positive or pleasant experience (10%; n = 3) and made them feel confident/safe in the care provided (10%; n = 3).

### Cross-checking and cognitive artefacts supporting maternity handover

The ability to cross-check information was mentioned by 16 participants (53%). There was reference to the patient being involved in the cross-checking (43%, n = 13) and of observed direct inter-professional cross-checking (10%, n = 3).

Participants described the use of articles to assist cognition and memory [26] in maternity handover and clinical communication through their interviews. The most frequently mentioned cognitive artefact was patient authored birth plans (50%, n = 15), followed by medical records including patient charts (27%, n = 8).

The cross-checking of the cardiotocograph (CTG) was reported by two participants. Cross-checking of items (e.g. CTG interpretation) with another clinician was regarded by one participant to be good professional practice.

The medical record was used in a range of locations in pregnancy care (antenatal clinic, shared antenatal care with the community general practitioner, birth suite, transfers between departments and the ward) whereas the patient authored birth plan was exclusively referred to in the birth suite.

Cognitive artefacts were perceived to support handover by being an archival form of delayed handover, as prompting memory during handover, or as a means to facilitate the cross-checking of information for accuracy, detail or to fill in perceived gaps as required. Although all participants were English-speaking, one believed that officially documenting clinical information to support handover may be particularly important for non-English speaking patients (who were excluded from the current study).

Some participants felt that certain pieces of information were important to both officially document in their medical record and verbally handover (20%; n = 6), such as relevant medical history (e.g. substance dependence), preferences about Konakion (Vitamin K) for the newborn, preferences regarding episiotomy, or requirement for an interpreter.

Participants also observed clinicians making distinctions about information that should be documented as well as verbally handed over. One woman described how a plan for her management was documented, shown and discussed with her by a clinician, in addition to being verbally handed over which she considered good practice. Consistency in management was important to her.

The most frequently reported cognitive artefact was the patient-authored Birth Plan (50%; n = 15). As one participant said, "I orchestrated my own handover." When a Birth Plan existed, some patients were happy for just an awareness of its existence (30%; n = 9) or just the relevant parts (10%; n = 3) to be verbally handed over as it could be checked for detail later (17%; n = 5). One participant did not have a written Birth Plan, but had developed a verbal Birth Plan with her husband and felt it was his duty to make her wishes known in labour.

Although cognitive artefacts by design may be used to enhance the quality and safety of maternity handover through assisting memory and cognition, participants described incidents in their care where there were problems. For two patients where documented patient-authored Birth Plans were present, points that the patient believed were important were perceived to be not recognised or handed over by clinicians. These women described instances: 1) where there was a failure to provide analgesia (nitrous gas or pethidine) in a timely manner when requested by the patient, and 2) when a patient's partner was not offered the opportunity to cut the umbilical cord at birth without an explanation given as to why this was not possible. One of these patients however offered suggestions to avoid similar incidents in the future. Her suggestions were twofold: placing the patient-authored Birth Plan in a visible place, such as being stuck to the door, and dedicating a session during Childbirth Education to Birth Plans. She also suggested that the hospital offer interested patients standardised templates for patient-authored Birth Plans. The other affected patient wondered whether the lack of consideration of her Birth Plan might reflect how different Birth Suites (with different models of care) attributed different levels of importance to patient-authored Birth Plans. This patient had been transferred from a lower-risk, midwifery-care Birth Suite to a higher-risk Birth Suite intra-partum when this event occurred.

Formal antenatal hand-held records were perceived to be ineffective in supporting handover and clinical communication by one patient who had received shared antenatal care. Her observation was that the hospital clinicians and the general practitioner did not read notes from previous visits with the other service at each of her visits. In particular, she felt rushed in the hospital clinic which she perceived may have contributed to clinicians not reading previous notes in her handheld record. This experience made her consider having all her care within the hospital for her next pregnancy, as she had observed good team work and shared awareness of clinical information between hospital clinicians.

### Participation of patients and support people influencing the quality and safety maternity care

Within the interviews, a common theme referred to patients and their support people's involvement in maternity handover and care, and how that impacted on quality and safety. In the Birth Suite setting in particular, over half of the participants preferred handover to occur in their presence inside the Birth Suite room. For a few, this was influenced by the stage of labour. In particular, five women did not wish to be present at handover in second stage (17%; n = 5) so not to distract or distress them at that time (13%; n = 4).

The ability of the patient to clarify, add, validate and update information at the time of handover was described (37%; n = 11), with one patient commenting that "patients often have more information than you [clinicians] anticipate." If the patient was not present during handover, the ability to supplement, clarify and update information later in the shift was also felt to be important (23%; n = 7). Particular information that women believed should be cross-checked was their preference for episiotomy, syntocinon for third stage management, Hepatitis B vaccination and/or vitamin K injection for the newborn, as well as whether clinical issues that were handed over between clinicians were still a problem. Two patients also commented that it was the support person's role to make patient wishes known and be involved in the cross-checking of the patient-authored Birth Plan with clinicians during labour; one observed their support person (husband) in the Birth Suite being involved in handover and believed it was the clinicians' duty to facilitate this involvement.

Some participants reported they had the right to know information that was handed over and discussed (13%; n = 4). A couple specifically described the importance of being involved in discussions that took place in their presence (7%; n = 2). Two women commented that staff had included them in their pregnancy care and cited that patient involvement was encouraged by the avoidance of technical jargon/acronyms (7%; n = 2) or being patronising in their discussion (3%; n = 1).

Understanding of current management decisions (10%; n = 3) was an important factor for some when the management plan was constantly changing (7%; n = 2). This regularly happens during labour, impacting on patient-authored Birth Plans, as one woman had mentioned. Similarly, to participate in shared decision making was considered reassuring or useful by the patient (10%; n = 3). Some participants felt that they did not always need to be present during handover in order to have input; they specifically identified ways in which this input could occur: including information from prior patient-clinician discussions (13%; n = 4), referring to patient-authored Birth Plans (50%; n = 15); and consideration of patient's emotional state in labour, such as coping and distress (23%; n = 7). In this way, participants felt that they continued to have an impact on decision-making, despite their physical absence.

However, not all women were consistently happy to take a role in enhancing the safety and quality of handover. Eight women (27%) specifically did not wish to be present in handover during labour. Six women (20%) expressed feelings of vulnerability when asked about the concept of being present and/or involved with handover in the labour setting. One woman when asked by a clinician why a particular investigation was being done, wondered why this information was not handed over, either verbally or through documentation, and therefore questioned whether she needed to have it done at all. Similarly, another woman felt that clinicians' expertise and authority were undermined if they asked their patients about information or decisions which had been previously discussed with another clinician. In addition, four women (13%) felt that their presence at handovers during labour may negatively impact on the discussion taking place. Other negative effects mentioned were: increased handover time, hampered professional discussion, patient interruptions, or increased risk of panic though misunderstanding the information discussed; in particular, the latter point was perceived by some of our English speaking participants to be a problem for women with little or no English.

## Discussion

The participants in this study described patient-centred strategies to promote quality and safety through handover through TSA between clinicians as well as patients (and support people). This reflects the potential roles of the patient and her support people not only as the recipient of health care but also as an active participant in her own management.

Collaborative cross-checking of information with the support of cognitive artefacts was also seen to enhance these aspects of handover and communication. These strategies have been identified in the literature to promote teamwork and patient safety both within handover and in a wider context [27-33].

Although the direct voices of the patients' support people are not heard through these interviews, it is known that support people wish to feel involved in the labour, be consulted (to be given spontaneous verbal information from clinicians and to be able to ask questions) and have demonstrated active preparation to undertake this role in the birth suite. Their involvement was related to their satisfaction with the birth experience including their feeling of being able to fulfil their planned supporting role [34,35]. This echoes the participants reporting the important role they expected of their support people during labour and is a question for exploration in future research.

Novel in this study is the identification by participants of the patient-authored Birth Plan as a cognitive artefact that plays an important role in maternity handover and was discussed by half the sample. Their existence has been described previously as a means to open up general channels of communication between clinicians and patients allowing negotiation of conflicting management or expectations [25,36], rather than a strategy for enhancing quality and safety. Benefits described included an improved clinical understanding and knowledge of choices during labour and childbirth, as well as assisting women (and their partners) to express their needs and preferences during that time [36-38]. This was particularly important for those who perceived a decreased ability to do this due to the pain or stress experienced during labour [37,38]. In our study, some patients expressed a wish to be consulted when management plans changed, particularly those which impacted on or were contrary to patient-authored Birth Plans. This consultation process is important as women reported feeling increased stress at times when management diverged from their original documented plan [37]; this stress was felt to be potentially allayed by ongoing consultation.

At the same time however, it is important to recognise that patients vary in their preferences for involvement in the 'medical work' of handover. Almost a third preferred not to be present at handover during labour. In particular, some felt it may negatively impact on the quality and the safety of the process.

The hand-held record was an additional identified cognitive artefact. Hand-held antenatal medical records are common tools of communication in shared care arrangements [39], particularly when clinicians are located in geographically disparate settings. Benefits reported by patients in a randomised control trial of hand-held records identified included feelings of increased confidence, personal responsibility and control over their pregnancy; they were also seen to facilitate better communication between women and health professionals [40]. In addition, in the case of patient-authored birth plans, these less formalised hand-held records were seen to enhance communication with, and advocacy by, support persons. Our participants described the importance of this communication tool to represent their voice and/or views within handover. In this way, formal antenatal handheld records and patient-authored birth plans facilitated a sense of TSA between clinicians, patients and support people. When combined with their reports of feeling fully informed and motivated towards information-seeking and self-education during pregnancy, hand-held records also facilitated women's understandings about their condition. Our findings echo these, and highlight the role of the hand-held record as a keystone to enhancing quality and safety in maternity care. This also reflects their capacity to further facilitate cross-checking at a later stage in care, as well as reassuring and motivating the patient during her labour and enhancing her confidence in her maternity care.

At the same time, however, some of the supportive human factors identified for handover appeared to degrade the system when the context was changed. What one patient perceived as enhancing quality and safety of handover was sometimes seen as having the opposite effect for others. As we identified in this paper, some patients experienced distress when unwillingly involved in bedside handover; other patients perceived this inclusion positively. In addition, context was changed by the actions of clinicians. Participants cited the use of technical language (jargon) that excluded patient understanding as undermining their contribution to bedside handover; this was thus was seen to degrade quality and safety. Differential levels of importance placed on cognitive artefacts, particularly patient-authored Birth Plans, which were either not acknowledged or used ineffectively in some cases, further undermined the positive effects of handover described by participants.

### Limitations of the study

The decision by the researchers not to audio record interviews was a significant limitation of the current study, which may have led to loss of the more nuanced data and contributed to recall bias on the part of the interviewer when writing up the account of the interview afterwards. We attempted to minimize the impact of this during the interviews in two ways: firstly, the interviewer repeated points back to the patient in order to check that she clearly understood what the patient was saying; secondly, the interviewer sought the patient's clarification of any ambiguous responses during the interview. Note taking at the time of the interview also ensured that important data was retained, and was expanded immediately afterwards in an attempt to counter the effects of recall bias.

The demographic profile of our participants limits the generalizability of our findings as over half of the people who were approached declined to participate. The majority of the interviewed sample had completed secondary or higher education. The effect of under-representation of participants with less formal education is unknown and thus we are unable to comment on their experiences or views. Other groups not directly studied were patients under 18 years, non-English-speaking patients and support people. Perceptions of these under-represented groups are a possible direction for future maternity handover research.

## Conclusions

There is a patient awareness of maternity handover. Some of them view evidence of handover, through clinician awareness of information, as representing positive aspects of teamwork, care and communication.

When patients were aware of handover, they observed that cross-checking and cognitive artefacts could be used to enhance the quality and safety of this process. Positive patient involvement in cross-checking of information and communication to support handover were described by some. Indirect patient involvement in handover through patient-authored birth plans was described by a number of participants. This was a novel finding.

This study suggests that there is merit in further exploration of involvement of patient and support people's role in handover and the evaluation of improvements to this process in the future.

## Competing interests

GSMC and LK were employed by the organisation where the participants of this study were receiving care during the course of the study. Neither author was involved in the direct care of the patients studied.

## Authors' contributions

GSMC participated in the conception and design of the study, conducted the interviews and data analysis, and drafted the manuscript. NW participated in the data analysis and drafted the manuscript. LK and PC participated in the conception and design of the study. This manuscript reports on the findings of GSMC's doctoral research, which was supervised by the other three authors. All authors read and approved the final manuscript.

## Pre-publication history

The pre-publication history for this paper can be accessed here:

http://www.biomedcentral.com/1471-2393/11/58/prepub
